# Digital Scientific Platform for Independent Content in Neurology: Rigorous Quality Guideline Development and Implementation

**DOI:** 10.2196/35698

**Published:** 2022-06-09

**Authors:** Daniel Kantor, Martin Farlow, Albert Ludolph, Joan Montaner, Raman Sankar, Robert N Sawyer Jr, Fabrizio Stocchi, Agnès Lara, Sarah Clark, Karine Deschet, Loucif Ouyahia, Yacine Hadjiat

**Affiliations:** 1 Florida Atlantic University Boca Raton, FL United States; 2 Nova Southeastern University Fort Lauderdale, FL United States; 3 Department of Neurology School of Medicine Indiana University Indianapolis, IN United States; 4 Department of Neurology University of Ulm Ulm Germany; 5 Deutsches Zentrum für Neurodegenerative Erkrankungen Ulm Germany; 6 Department of Neurology Hospital Universitario Virgen Macarena Seville Spain; 7 Division of Neurology Department of Pediatrics UCLA Mattel Children's Hospital Los Angeles, CA United States; 8 Department of Neurology University at Buffalo State University of New York New York, NY United States; 9 University San Raffaele Roma Rome Italy; 10 Istituto di Ricovero e Cura a Carattere Scientifico San Raffaele Roma Italy; 11 Medicom Concept Occitanie France; 12 Biogen Digital Health Cambridge, MA United States

**Keywords:** digital health, Neurodiem, neurology, neuroscience, eHealth, methodology, content quality, guideline, platform, development, implementation, quality, brain, communication, health information, health care professional, assessment, monitoring

## Abstract

**Background:**

Digital communication has emerged as a major source of scientific and medical information for health care professionals. There is a need to set up an effective and reliable methodology to assess and monitor the quality of content that is published on the internet.

**Objective:**

The aim of this project was to develop content quality guidelines for Neurodiem, an independent scientific information platform dedicated to neurology for health care professionals and neuroscientists. These content quality guidelines are intended to be used by (1) content providers as a framework to meet content quality standards and (2) reviewers as a tool for analyzing and scoring quality of content.

**Methods:**

Specific scientific criteria were designed using a 5-point scale to measure the quality of curated and original content published on the website: for Summaries, (1) source reliability and topic relevance for neurologists, (2) structure, and (3) scientific and didactic value; for Congress highlights, (1) relevance of congress selection, (2) congress coverage based on the original program, and (3) scientific and didactic value of individual abstracts; for Expert points of view and talks, (1) credibility (authorship) and topic relevance for neurologists, (2) scientific and didactic value, and (3) reliability (references) and format. The criteria were utilized on a monthly basis and endorsed by an independent scientific committee of widely recognized medical experts in neurology.

**Results:**

Summary content quality for the 3 domains (reliability and relevance, structure, and scientific and didactic value) increased in the second month after the implementation of the guidelines. The domain *scientific and didactic value* had a mean score of 8.20/10. Scores for the domains *reliability and relevance* (8-9/10) and *structure* (45-55/60) showed that the maintenance of these 2 quality items over time was more challenging. Talks (either in the format of interviews or slide deck–supported scientific presentations) and expert point of view demonstrated high quality after the implementation of the content quality guidelines that was maintained over time (15-25/25).

**Conclusions:**

Our findings support that content quality guidelines provide both (1) a reliable framework for generating independent high-quality content that addresses the educational needs of neurologists and (2) are an objective evaluation tool for improving and maintaining scientific quality level. The use of these criteria and this scoring system could serve as a standard and reference to build an editorial strategy and review process for any medical news or platforms.

## Introduction

Over the last three decades, digital communication has emerged as a major source of medical information for health care professionals in a wide range of specialties, including neurology [1-3]. Web-based medical content is a valuable resource to provide updates on the latest medical news, share information with peers, and support education [4,5]. In light of the rapid growth in the number of medical websites with various content streams, either curated or original, the quality of scientific information that is disseminated through these tools has become a critical issue [6,7]. Thus, there is a need to ensure that digitally published scientific information dedicated to health care professionals is accurate, credible, relevant, and unbiased.

No single standard exists to objectively evaluate the quality of medical information available on the internet [8], although frameworks and measurement tools that provide users, including clinicians and researchers, with quality assessment options when navigating on an information platform have been proposed [9-17]. A simple and reliable methodology for screening and assessing the quality of web-based information could be a first step in developing optimal editorial guidelines that can be applied to digital scientific content and allow the production and sharing of reliable and accurate materials for the medical community A quality process for web-based medical communication should be equivalent to those established for peer-reviewed scientific journals and include scoring grids of criteria to assess the quality, originality, and relevance of published content.

Neurodiem (Biogen Inc) is a free multilingual and multicountry digital platform for independent information and education—the latest news and literature in neuroscience—dedicated to health care professionals and scientists Neurodiem is nonpromotional and provides strictly independent, impartial, and unbiased scientific content (in particular, with respect to Biogen’s drug portfolio and therapeutic areas of interest). To meet this requirement, content published on Neurodiem is selected or generated exclusively by third-party publishers based upon the advice of advisory committees of neurologists and the published information covers all subspecialties of neurology in a balanced manner.

In addition, content on Neurodiem is monitored by a scientific steering committee of expert neurologists (who oversaw the development of the platform and who contributed to the development of these content quality guidelines).

The platform publishes content such as (1) summaries of curated peer-reviewed journal papers (extractions of the most relevant information from a published paper), (2) coverage of and comments on communications from neurology conferences, and (3) presentations and talks on currently debated topics by expert neurologists, and also provides (4) access to a selection of full-text papers from top-tier journals in neuroscience.

The Neurodiem steering committee developed a robust approach to evaluate scientific quality standards for content, while maintaining the independence of delivered information—scientific and medical content quality guidelines for Neurodiem content were first released in January 2020 (Multimedia Appendix 1) and described not only governance, roadmap and workflows for the quality process, but also, proposed custom criteria and a scoring system for the analysis and monitoring of scientific and medical various content stream quality.

The objective of this paper is to respond to an unmet need for content quality control with a new methodology for the assessment of platform content quality.

## Methods

### Content Quality Criteria and Rating System

The Neurodiem steering committee developed content quality guidelines in order to maintain and improve the quality of scientific content published on the website. We shared these content quality guidelines with stakeholders involved in the content quality process. First, content providers required specifications for scientific and medical quality standards adapted to and expected by a neurology audience, and second, scientific reviewers received an objective quality criteria and rating system which identifies areas of improvement, which can be shared with content providers on an ongoing basis.

A grid with specific quality criteria was designed for each content stream—summaries, congress highlights, expert points of view, and expert talks—published on Neurodiem (except for licensed content, ie, full-text journal papers that have already gone through a peer-review process).

Quality criteria were defined and organized into domains to support granular analysis of content quality based on scientific relevance as well as editorial and journalistic standards featuring information dedicated to a specialized health care professional audience.

A 5-point scale from 1 (lowest score) to 5 (highest score) was used to rate each criterion. Each quality domain rating was multiplied by a criticality coefficient, and the products were summed for the total quality score; each domain was validated by the steering committee. This quality evaluation method was developed using the Neurodiem platform as an example, and it is most appropriate to Neurodiem content and is specific to each section of the platform. Using generalized evaluation grids could be less accurate and not applicable for all sections, which is why a new flexible method was needed for Neurodiem and similar platforms.

### Review Process

In order to analyze and rate the scientific quality of Neurodiem content, 4 countries (France, Germany, Spain, and Italy) were selected for pilot implementation of the content quality guidelines for a 1-year duration. Reviewers had to (1) be a native speaker of a language represented on the Neurodiem local platform, (2) have a high-level scientific and medical profile (including a solid background in neuroscience or neurology), (3) have experience writing, reviewing, and editing scientific content, and (4) commit to a minimum of 1 year, in order to ensure content review homogeneity and be able to perform long-term assessment of the process.

In order to avoid any influence of the review process on content selection and production, the review process was performed after publication (usually within 1 month). Scientific content quality was assessed on a monthly basis for 1 year (10 months; no reviews were performed in August and December).

Due to the high volume of content published on Neurodiem, the quality review process was only performed on a representative sample—papers and talks were selected from the 18 neurology topics on Neurodiem (cognition, critical care, dementia, epilepsy, genetics, headache, imaging, movement disorders, multiple sclerosis, neuro-oncology, neuro-ophthalmology, neuromuscular, neurosurgery, pain, pediatric neurology, rehabilitation, sleep, and stroke) for comprehensive and balanced coverage. The content submitted to review was selected either randomly or if subject matter experts identified the topic as particularly challenging from a scientific accuracy or complexity perspective (eg, cutting-edge imaging, biotechnology, genetics-related content).

We monitored the quality of content published on the Neurodiem website over a 1-year period. The defined target for the monitoring rate was 20% of published content. The review process for Summary content was carried out by a single reviewer to ensure evaluation homogeneity over the period. Baseline data (month 1) were collected when Neurodiem content quality guidelines were not yet in place. The content quality guidelines were implemented in month 2, and analytics and quality improvement objectives were shared with content providers on a monthly basis.

### Methodology Endorsement by a Scientific Committee

After 1 year, an independent committee, which consisted of 8 international neurology experts (based on experience and subspecialties in neurology, willingness to work in a digital field, experience using Neurodiem, and their availability) from Germany, Italy, Spain, Canada, and the United States, was involved in providing guidance and evaluating the scientific validity of the quality review process. The experts could not be involved in any commercial activities with Biogen over their period of engagement (to ensure their independence from the project sponsor). The scientific committee members were asked to review selected sections of Neurodiem content quality guidelines (dementia, epilepsy, movement disorders, multiple sclerosis, neuromuscular disorders and rare diseases, neurovascular diseases, and pediatric neurology), and then, were individually interviewed to ascertain their feedback and suggestions. Neurologists’ advice and proposals related to definitions, wording, and validity of quality criteria, as well as scoring used for rating, were synthesized in a group meeting. An updated version of the Neurodiem content quality guidelines was released in January 2021 (Multimedia Appendix 1).

### Scientific Quality Specifications and Measurement Tool

#### Summaries

##### Overview

Paper summaries cover curated full-text papers published in peer-reviewed journals in the field of neuroscience. The scientific quality of a summary depends on the accurate and succinct articulation of the content from the source paper in line with the predefined format for this content type (Multimedia Appendix 1). Three quality domains were defined: (1) reliability and relevance (of the source content and of the topic) with respect to an audience comprising neurologists, (2) structure of the summary, and (3) the scientific and didactic value of the summary.

##### Reliability and Relevance

Criteria are listed in Table 1.

**Table 1:** Reliability and relevance criteria.

Item	Criterion description
Journal	Papers curated for generating summaries should be originally published in high-impact factor or renowned peer-reviewed journals in neurology or neuroscience, to target scientific information primarily validated by a board of editors and reviewers. The journal quality assessment is based on H-index classification used in the field of Clinical Neurology, which was recommended by the scientific steering committee. The score reflects a neurologist’s quality assessment of the journal: 1 for a paper not curated from a peer-reviewed scientific journal; 2 for a journal not classified in the SCImago Journal Rank; 3 for H-index values <30, 4 for H-index values 30-69, and 5 for H-index values ≥70.
Topic	Selected papers should be representative of current and major and scientific news at the forefront of information in each neurology subspecialty. According to the needs and interests of Neurodiem audience, selected topics should preferentially have direct impact on clinical practice or translate into major changes of the research and development landscape in neurology. The topic is scored on a scale from 1 to 5, based on the contribution in the neurology field or direct or the immediate impact in the clinical practice based on the author’s conclusion.

##### Structure

Items in the *structure* domain (Table 2) were given a score between 1 (strongly disagree) and 5 (strongly agree), based on accuracy and the informative nature, ability to be understood, and attractive value of the original paper’s content. Item scores were summed to generate a domain score out of 25 (Multimedia Appendix 1).

**Table 2:** Structure criteria.

Item and subitems			Description
Title and teaser text			The title and teaser text are the entry points to the paper summary on the home page and, thus, require particular attention. These 2 elements were evaluated on the basis of accuracy of the information, attractivity, clarity, and conciseness. The title should reflect the actual and main findings of the original paper. The teaser text should be distinct from the title text and provide more information while leaving the readers curiosity opened to explore the paper.
Take away			This section should contain 1 to 2 sentences to summarize the main findings of the source paper. Considered to be independent from the rest of the summary, this section is evaluated according to the clarity and relevance of the main results supporting the authors’ conclusions.
Why this matters			In the format of 2 bullet points, this section is evaluated based on whether or not the structure and information on the clinical practice included in the original paper are respected. This section should (1) provide contextual information about the state of the art prior to the study and why it was interesting to explore the subject and (2) highlight study results’ critical clinical implications or impact, in terms of disease mechanisms or pathophysiological paradigm changes, candidate molecule development, anticipated switch of clinical practices and content quality guidelines, in neuroscience.
**Study design**			This section is evaluated based on whether the main material and methods used in the study, focused on the key elements in relation to the study results, are summarized, complete and accurate. Layout features for this paragraph should imperatively include
	Study objective	The primary endpoint; when relevant, the secondary endpoints The characteristics and size of the analyzed population, subpopulations, if applicable; animal model of pathologies will also be defined if needed The study design, in particular, groups being compared The follow-up duration and critical time points of analysis The description of the procedures, clinical scales, or parameters being measured as well as the rationale of these measurements (ie, the expected outcomes. Synthetic background information on investigations performed may be provided when dealing with cutting-edge technologies not obviously known by any subspecialists in neurology)	
	Key results	The *Key results* section should provide an adequate and concise description of the major findings of the work. Primary endpoint–related results should be prioritized. Secondary endpoints may be included when extending the field of knowledge or paving the way for new hypotheses to be tested. It is important to illustrate remarkable results by providing numerical data with exact *P* values together with confidence intervals to illustrate the effect size and clinical value of data. It is recommended that the study’s key take-home messages, including medical interpretations, be summarized at the end of this section in order to facilitate the retention of this information by readers.	
	Limitations	The *Limitations* section should briefly summarize study methodological characteristics potentially impacting findings interpretation and usually addressed in the discussion part of the source paper.	

##### Scientific and Didactic Value

The domain *scientific and didactic* comprises 2 items (Table 3) scored from 1 (strongly disagree) to 5 (strongly agree), which were summed to generate the domain score (Multimedia Appendix 1).

**Table 3:** Scientific and didactic value criteria.

Item	Description
Accuracy	The paper summary must represent the original curated content with (1) accurate scientific glossary, abbreviations, and the numerical and statistical data described in the original full-text paper; (2) the accurate and relevant summary of the methodology, results, interpretation, conclusion, and impact in the clinical setting of the original full-text paper.
Didactic dimension	The didactic dimension is evaluated based on whether or not the Summary is clear, succinct, and comprehensible at first reading. It is important to provide enough background information, including context and scientific and medical definitions that are not common or shared among the neurologist community (eg, gene and protein functions, mode of action of new molecules, expected outcomes from emerging technologies). Understandability and readability of the Summary should be also supported by critical data that are presented logically and coherently.

##### Overall Quality

To obtain the overall quality score for paper Summaries, each domain subscore was weighted by a coefficient: 1, for reliability and relevance; 2, for structure; and 4, for scientific and didactic value. The products were summed to generate a total score out of 100.

#### Congress Highlights

##### Overview

Congress highlights present coverage of posters or oral communications from international and national top-tier conferences in neurology in an abstract format. The Neurodiem editorial team proposes a mean coverage of 1 conference per month.

Quality domains considered for reviewing Congress highlights include (1) congress selection relevance, (2) topic selection, and (3) the scientific quality of generated abstracts.

##### Congress Selection Relevance

This content covers the main international and national conferences in the neurology field. The congress should address topics related to one or multiple subspecialties in neurology (scored out of 5, where 0 is not relevant and 5 is highly relevant).

##### Congress Coverage

The objective of congress coverage is to provide medical news that faithfully reflects the original congress’ program, spirit, and potential scientific and medical breakthroughs. Thus, congress highlights should be characterized by 3 criteria (Textbox 1). Each criterion received a score out of 5; scores were summed for an overall congress coverage score out of 15.

**Coverage criteria.**

**Criteria**

- Coverage of both scientific and clinical-oriented topics. According to the clinician audience targeted for Neurodiem, selected topics should have a direct impact on clinical practice or translate at some point into clinical development or evolution in clinical practice.
- Coverage of hot topics, scientific or clinical highlights and late-breaking news sessions, representative of major and most topics expected to be presented during the congress.
- Coverage of both posters and oral communications, prioritizing oral communication with more validated and impactful outcomes (no more than 10 or 15 per 100 posters).

#### Scientific and Didactic Value (Individual Abstracts)

##### Structure

The *structure* domain for conference abstracts is similar to that for summaries. In addition, it is recommended that a short comment from an expert neurologist that identifies implications for clinical practice and clinical research milestones achieved or to be further defined be included.

##### Accuracy and Didactic Dimension

When applicable, scientific quality assessment should be based upon whether the main scientific content of the original congress communications was respected.

The didactic dimension of the abstract should be assessed on the ability to highlight new concepts and translate the findings into clinical practice.

Each item is scored out of 5, from 1 (strongly disagree) to 5 (strongly agree); scores were summed for an overall domain score out of 10.

##### Overall Quality

Each domain subscore was weighted by a coefficient: 1, for congress selection relevance; 2, for congress coverage; 1, for structure; 4, for accuracy and didactic dimension; the products were summed to generate a total score out of 100 (Multimedia Appendix 1).

#### Expert Points of View and Talks

##### Overview

Expert points of view and talks were developed by the Neurodiem editorial team exclusively for this platform in order to offer a synthesis on a current neurology topic by a recognized medical expert in the field. Expert points of view and expert talks are intended to offer a synthesis on a neurology current topic by a recognized medical expert in the field to allow neurologists to get expert opinions or overviews on emerging, state-of-the-art, or hot topics in neurology. Expert points of view and talks were assessed by an independent reviewer.

Subscores for 3 quality domains were weighted: a coefficient of 3 for credibility and relevance, a coefficient of 4 for scientific and didactic value, and a coefficient of 1 for reliability and format; the products were summed to generate a total score out of 100.

##### Credibility and Relevance

Authors or speakers and topic (Table 4) scores were summed to generate a domain subscore out of 10, which was weighted by a coefficient of 3.

**Table 4:** Credibility and relevance criteria.

Item	Description
Authors or speakers	Authors or speakers who have been selected to share their expert point of view should be key medical experts in neurology subspecialties (neurologist or neuroscientist). Presenters should meet quality standards in terms of academic seniority (Assistant Professor degree or equivalent), reputation among their peers and long experience (score out of 5; on a scale from 1, strongly disagree, to 5, strongly agree).
Topic	The topic should be related to recent advances or debated issues in the neurology or neuroscience. The subject should be of interest to the neurologist community; hence, content should have a valuable and original contribution to the field and a significant clinical impact (scored out of 5; 1, not related to neurology; 5, relevant and is a major contribution to the field).

##### Scientific and Didactic Value

Structure, accuracy and didactic dimension, and writing or speech quality (Table 5) were each assessed out of 5, on a scale from 1 (strongly disagree) to 5 (strongly agree), and summed for a domain subscore out of 15, which was then weighted by a coefficient of 4.

**Table 5:** Scientific and didactic value.

Item	Description
Structure	Structure for expert points of view and talks presentations were evaluated with a score from 1 to 5 based on the inclusion, accuracy, and the chronology order of An introduction that includes (1) scientific background information, (2) a rationale for topic selection based the current state of scientific and clinical knowledge, and (3) a presentation overview Scientific and clinical evidence supporting the topic including numerical key data An overview of why these results have a scientific and medical impact in the neuroscience/neurology landscape A summary of take-home messages and conclusions relating to anticipated milestones in neuroscience research, direct implications for clinical practice and/or updates to this content quality guidelines Importantly, the expert point-of-view structure will be supported by occurrence of relevant and meaningful subheadings for each paper’s section or by titles corresponding to the different sections or main messages of the presentation. Overall, attention should be paid to logical development of scientific arguments and evidence.
Accuracy and didactic dimension	Accurate and concise background information and research or clinical context Relevant selection of specific arguments, scientific evidence, and illustrations for supporting expert demonstration
Writing or speech quality	Authors or speakers should display the ability to synthesize ideas and provide simplified explanations of cutting-edge techniques or complex concepts Writing or speech style: a neutral, factual, and formal tone should be used Clarity and coherence: logical links between arguments and sections supporting scientific discussion The quality of English or local language and grammar should be appropriate

##### Reliability and Format

Each of these 2 items (Table 6) were scored out of 5 based on duration of the presentation (1, for too long or too short in duration; 5, strongly agree for those approximately 5 minutes) and summed to generate a domain subscore out of 10 (Multimedia Appendix 1).

**Table 6:** Reliability and format.

Item	Description
References	References cited in expert point of view or talks should be focused, and source of references should be reliable and consequently selected exclusively from (1) high-impact factor or recognized peer-reviewed journals in the neurology or neuroscience field (2) validated and up-to-date clinical guidelines.
Format	Owing to the summarized format targeted for expert point of view, core content of the paper should not exceed 1500 words. Likewise, informal talks should not exceed 5 minutes (to respect technical feasibility because big files cannot be uploaded to the platform), audience expectations (scientific community has short time to watch presentations and videos)) while more academic presentations should be between 5 and 10 minutes.

## Results

### Summaries

**Figure 1:**
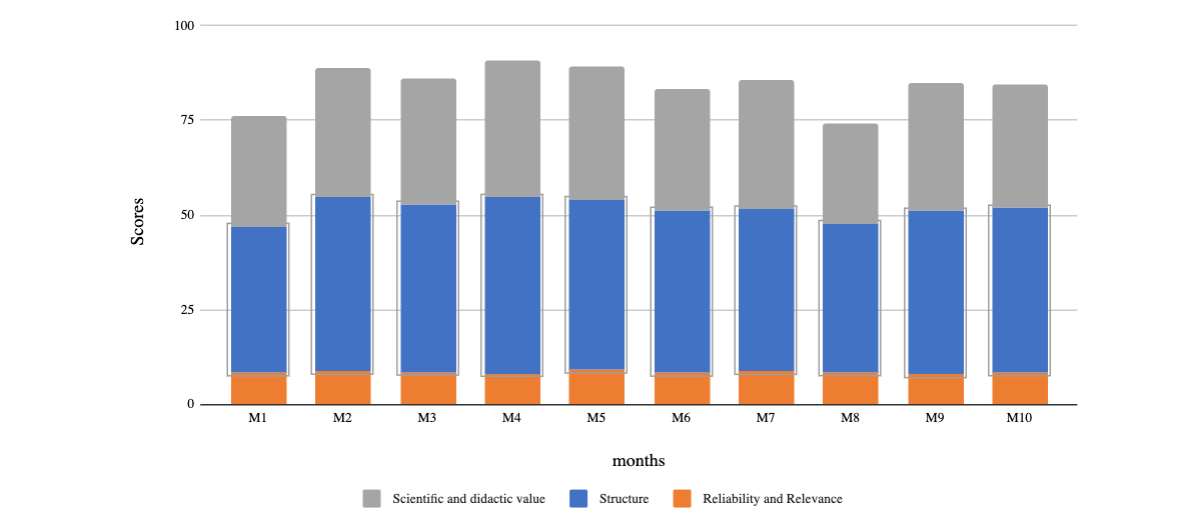
Analysis and monitoring of scientific quality applied to Summaries published on Neurodiem over a 12-month period.

The overall quality score increased from 75 in month 1 to 85 in month 2. The scores stabilized at a high level (score range between 70 and 80/100) (Figure 1). The domain *scientific and didactic value* had a mean score of 8.20/10.Those for *reliability and relevance* (8-9/10) and *structure* (45-55/60) showed that maintenance of these quality items over time was more challenging.

**Figure 2:**
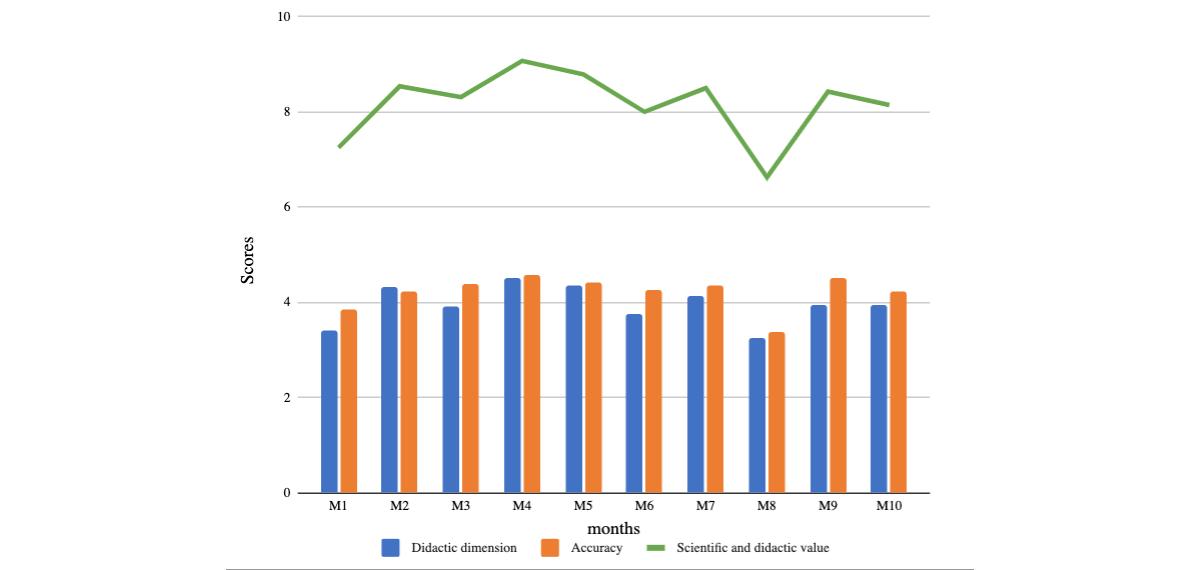
Analysis and monitoring of scientific and didactical quality applied to article Summaries published on Neurodiem over a 12-month period.

The ratings for the domain *scientific and didactic value* for the 12-month analysis period showed a trend similar to that of the structure domain an increase until month 4, followed by steady state, with a brief drop at month 8 (Figure 2).

### Expert Points of View and Talks

**Figure 3:**
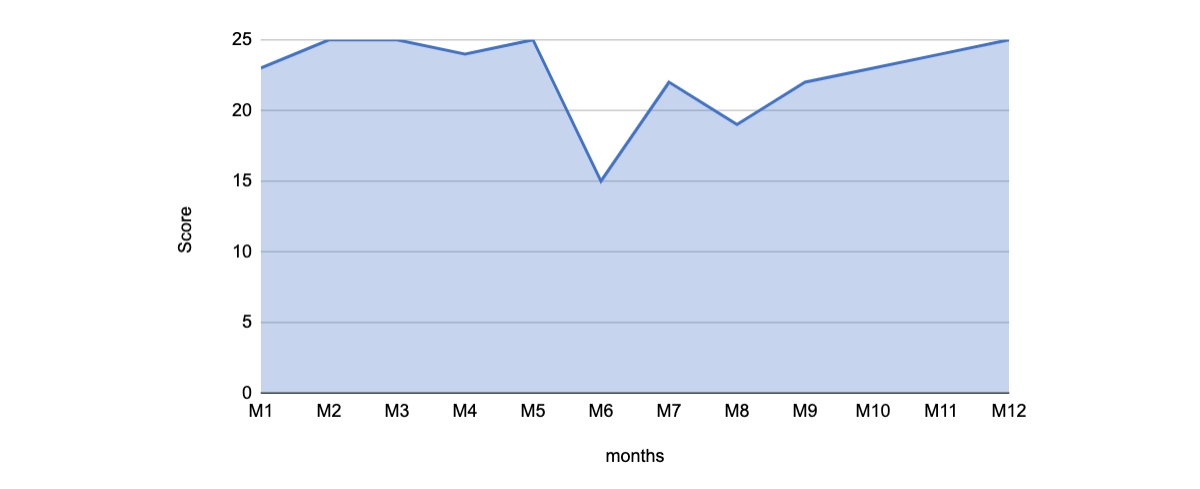
Analysis and monitoring of scientific and didactical quality applied to Expert points of view published on Neurodiem over a 12-month period.

Talks were always provided by medical experts without revisions or changes and were maintained in their original format for publication in Neurodiem Scores were high, which was sustained over time (Figure 3). Similar to those for expert talks, scores for expert point of view were high, which was sustained throughout the 12-month period.

## Discussion

These evaluation scores and criteria showed that the chosen methodology was appropriate to evaluate content quality shared on internet scientific platform. The development and use of content quality guidelines, with editorial specifications and a measurement tool, ensure that the scientific and medical content on Neurodiem is high quality. The quality criteria and scoring system facilitate the delivery of relevant, reliable, and current information in neurology while respecting the editorial independence of third-parties and expert panel who are involved in the selection and production of content published on the website. This approach has multiple uses. This framework is intended for content providers and medical writers in order to define content quality guidelines and standards for reaching scientific and medical excellence of the content. The content quality guidelines provide a simple criteria checklist and evaluation scale to deliver an objective analysis and quantitative assessment of content, and monthly monitoring of papers and talks provides regular quality reports and objectives to content providers, which leads to the improvement of quality over time.

For the Summaries, a parallel evolution between the structure and Scientific and didactic value could be explained by overlapping assessment objectives of some quality items in both quality domains. Interestingly, the rating decrease at month 8 was observed to be concomitant to some turnover in the medical writers’ team of content providers. Hypothetically, the drop could be due to delay in application of the content quality guidelines by the medical writers who recently joined the medical writers’ team.

Several eHealth information quality evaluation tools exist to answer the needs of different profiles of internet resource users, including patients and health care professionals [18,19]. These tools have features that overlap with those in our approach, such as assessing the credibility of digital content through experts scientific trust value (authors, speakers), the content, and content reliability via source checking of validity and up-to-date references. The importance for the target audience and their scientific needs are also highly represented in the checklist of both quality measurement systems. The accuracy of scientific content is addressed through the analysis of the strength and value of the scientific evidence provided. Moreover, content readability, such as appropriate language use, clarity of expression, and the logical flow of arguments are common quality criteria in digital medical information evaluations. Although not formally assessed in our approach, disclosures and conflicts of interest are systematically displayed in the author section on Neurodiem to ensure the website's editorial independence. The didactic dimension is an original and key feature of the Neurodiem quality approach. Based upon feedback from the scientific steering committee, the weighting of didactic value was increased, in order to highlight the importance of strong clinical relevance for Neurodiem content.

Our content quality guidelines are used to evaluate of scientific and medical content dedicated to clinicians, and although it was developed for use on a neurology-specific platform, the tool could be easily translated to any medical specialty. In addition, the Neurodiem content criteria grids are adapted to the format of web-based content (the most important sections allowed higher scoring). This methodology could be used by content creators or providers to support the production and review of content and information published on web-based scientific platforms. Alternatively, these content quality guidelines could also be of value to the medical community as a rapid and effective method of appraising the quality of content when consulting medical education websites.

There are some limitations to our quality assessment system. First, although our quality measurement tool provides a relatively strict framework for an objective rating, its application is nevertheless likely subject to inter-reviewer variability (the score attributed to each section may vary from reviewer vision to another). The difference in scores attributed by each reviewer during their assessment may be particularly pronounced for the evaluation of items such as didactic dimension or quality of speech and writing, which can be quite subjective and linked directly to the reviewer own interpretation. In order to reduce heterogeneity in content quality assessment, we propose that there be a standardized training session for reviewers, aimed to educate them on the adequate and consistent use of the scoring system. Second, some global and general conferences in neurology (eg, European Academy of Neurology, American Academy of Neurology) are characterized by a substantial communication program with a broad panel of scientific and clinical topics in distinct neurology subspecialties; as a result, we must recognize that assessments of whether congress coverage on the website is faithful to the original congress program are challenging. Thus, while our system is well suited to granular review of scientific and medical content at the single paper or topic level, improvements are needed for the assessment of content with a breadth of topics, such as a congress program. In future iterations of the content quality guidelines, automation and artificial intelligence technology could address this issue [20-22].

Our content quality guidelines are an editorial and quality evaluation system for information on Neurodiem that was developed to preserve editorial independence. Our methodology consists of a simple and short set of criteria to be used by content providers or reviewers to objectively assess the scientific and medical excellence of content, with special emphasis on impact and applicability in clinical practice. This standardized approach could be used on any biomedical news and resource digital platforms beyond the initial scope of neurology. These content quality guidelines support the implementation of a content quality strategy in the content creation phase as well as in the review process, which is the cornerstone of a high-quality digital communication platform [23].

## Appendix

Multimedia Appendix 1 Content quality measurement guideline.
