# Supplementary material for: Digital Scientific Platform for Independent Content in Neurology: Rigorous Quality Guideline Development and Implementation
Source: Interact J Med Res. 2022 Jun 9;11(1):e35698. doi: 10.2196/35698 (PMC9227648; doi:10.2196/35698)
Supplement: Multimedia Appendix 1 [file ijmr_v11i1e35698_app1.pdf]

Neurodiem content quality measurement

1. Purpose

The objective of this document is to provide a scoring system to support Neurodiem content quality assessment. This document is intended to be distributed among internal and external stakeholders involved in reviewing and monitoring Neurodiem content towards smooth improvement and maintenance of translation and medical content quality as per BHS defined standards.

2. Scope

This quality measurement system applies to monitoring of criteria for translation and scientific quality for Neurodiem content as defined in section 3. of the document “Neurodiem content quality specifications”.

3. Assessment of quality standards application

3.1. Translation quality measurement

In order to perform monitoring of translation quality level and progression, qualification for 3 distinct groups of translation defaults, as per deviations to quality criteria depicted in section 3.1. of the document “Neurodiem content quality specifications” has been defined. These KPIs apply to any type of content stream subjected to translation, i.e. CSS, COH, IDS and Talks (videos) described in section 2.4. of the SOP “Neurodiem content quality control”.

- *Mild translation defaults:*

These include typos, spelling and grammar mistakes in local languages, when not resulting in any misunderstanding or misinterpretation, especially from a scientific point of view.

- *Moderate translation defaults*

These include inadequate wording and improper use of scientific/medical glossary, but when these do not result in any misunderstanding or misinterpretation, especially from a scientific point of view.

- *Severe translation defaults*

These include inadequate wording and improper use of scientific/medical glossary and when these result in any misunderstanding or misinterpretation, especially from a scientific point of view.

3.2. Scientific and medical quality measurement

In order to perform monitoring of scientific and medical quality level and progression, a quality measurement tool, based on a 5-point scale, has been set up to assess application of the quality criteria defined in section 3.2. of the document “Neurodiem content quality specifications”. Instructions supporting use of this scoring system is presented below for each content stream.

3.2.1. CSS quality score

Neurodiem content scientific quality criteria and scoring system for article summaries (CSS) are described below. 3 main quality domains “Reliability and relevance”, “Structure”, “Scientific and didactic value” have been defined. Each of them includes quality items which are rated on a 5-point scale according to achievement of the described criteria. Scores in the individual sections are summed to generate a sub-score related to each quality domain and that is further weighted by a coefficient

(indicated on the right upper hand side of the criteria grid). All sub-scores are summed to generate a total score of up to 100 corresponding to the overall quality of the CSS.
Table 1 provided in the appendix corresponds to the template of the scoring table used by reviewers when analyzing CSS scientific & medical quality.

Reliability and relevance (source and topic) (score x1)

Journal. *Hindex in the subject category "Neurology (clinical)" (Scimago journal & country rank (SJR))*
1 - Article not curated from a peer-reviewed scientific journal
2 - Article curated from a journal that is not classified in the SJR filtered in the categories "Neurology", or "Neurology (clinical)", or "Neuroscience", or "Cognitive neuroscience"
3 – Article curated from a journal classified in the SJR in the category "Neurology (clinical)", with an H-index ranging <30
4 - Article curated from a journal classified in the SJR in the category "Neurology (clinical)", with an H-index ranging (30-69)
5 - Article curated from a journal classified in the SJR in the category "Neurology (clinical)", with an H-index ranging (70-351)

Topic

1- Topic not related to the Neurology/Neuroscience field (off topic)
2- Topic in the neuroscience field, but not of specific interest for neurologists (poor contribution and impact in the field, or purely basic research issue with no interest to clinicians)
3- Topic being a relevant contribution in Neurology, but very far from any application in the clinical setting (e.g. advances in neurological disorders pathophysiologic mechanisms only)
4- Topic being a relevant and major contribution in the field, anticipated impact in the clinical practice as per author's conclusion
5- Topic being a relevant and major contribution in the field, with direct/immediate impact in the clinical practice as per author's conclusion

⇨ **Sub-score reliability and relevance: /10**

Structure (specifications of each section) (score x2)

Title and teaser text
1-Does not reflect main findings of the source article
2-Scientifically accurate as per source content but not understandable for any neurologist sub-specialist audience
3-Scientifically accurate and understandable but not informative as per main findings of the source article
4-Scientifically accurate, understandable and informative as per source content but not attractive from a journalistic perspective
5-Scientifically accurate, understandable, informative and attractive.

Take away
1-Does not reflect main findings and conclusion of the source article
(2-3) - Does partially reflect main findings of the source article and/or lack critical background information
(4-5) – Does entirely reflect main findings of the source article and provides background information, towards being an independent section from the rest of the summary

Why this matters?
1- Does not accurately describe clinical impact of the study results as per source article
(2-3)- Lacks either scientific & medical novelties specifically brought by the study or potential impact of the study in the clinical setting
(4-5) – Describe accurately and didactically scientific & medical novelties specifically brought by the study and potential impact of the study in the clinical setting

Study design

Considering the following items: study objective, endpoint(s), population/model characteristics, study design, study duration, procedures and investigations (description and rationale): are these elements present and accurately presented?

1- None of these items are represented or accurately described as per source content
(2-3) Only some of these items are represented or may be not accurately described and explained
(4-5) All of these items are represented and are accurately described and explained

Key results

1- Results described are inaccurate as per original article (e.g. may provide wrong or biased scientific or clinical conclusion as per original article)
(2-3)- Results described are scientifically accurate but partially reflect author's conclusion or size effect/clinical value of data are not enough illustrated (e.g. numerical data, *p*)
4- Results described scientifically accurate; clinical value well illustrated, but no editorial selection performed to highlight only major results as per author's conclusion
5- Results described scientifically accurate; clinical value well illustrated; selection among results performed to present main findings supporting author's conclusion.

⇨ **Subscore structure: /50**

Scientific and didactic value **(score x4)**

Accuracy

Overall, does the article summary restitute accurately: scientific glossary/abbreviations, numerical and statistical data, as well as all methodology, results, interpretation, conclusion and clinical impact of the study?

1- Strongly disagree
2- Disagree
3- Neutral
4- Agree
5- Strongly agree

Didactic dimension

Overall, does the article provide sufficient background information (study contextualization, scientific definition) toward being understandable by any neurologist profiles; is it written logically and coherently so that the reading can flow?

1- Strongly disagree
2- Disagree
3- Neutral
4- Agree
5- Strongly agree

⇨ **Subscore scientific and didactic value: /40**

Total score article summaries CSS (overall quality): /100

3.2.2. COH quality score

Neurodiem content scientific quality criteria and scoring system for "congress highlights" (COH) are described below. 3 main quality domains "Congress selection relevance", "Congress coverage", "Scientific and didactic value" have been defined.

Each of them includes quality items which are rated on a 5-point scale according to achievement of the described criteria. Scores in the individual sections are summed to generate a sub-score related to each quality domain, which is further weighted by a coefficient (indicated on the right upper hand side of the criteria grid). All sub-scores are summed to generate a total score of up to 100 corresponding to the overall quality of the congress highlights encompassing relevance of congress selection and topic coverage as well as mean scientific and didactic value of a representative panel of abstracts analyzed at individual level.
Table 2 provided in the appendix corresponds to the template of the scoring table used by reviewers when analyzing COH scientific & medical quality.

Congress selection relevance (as per target audience) **(score x1)**

Does the program of the selected congress address needs of neurologists whatever their specialty (general conference in Neurology) or is it representative of main conferences in the Neurology sub-specialty covered?

1- Strongly disagree
2- Disagree
3- Neutral
4- Agree
5- Strongly agree

⇨ **Subscore congress selection: /5**

Congress coverage (topics) **(score x2)**

Overall, does the topics coverage on the website offers a balanced and representative panel of the followings items as per the original congress program and spirit?

Posters versus oral communications?

1- Strongly disagree
2- Disagree
3- Neutral
4- Agree
5- Strongly agree

Scientific versus clinical-oriented topics?

1- Strongly disagree
2- Disagree
3- Neutral
4- Agree
5- Strongly agree

"hot topics", "scientific/clinical highlights", and "late breaking news" sessions?

1- Strongly disagree
2- Disagree
3- Neutral
4- Agree
5- Strongly agree

⇨ **Subscore congress coverage: /30**

Scientific and didactic value (individual abstracts)

- Apply scoring for the 5 items of the "*structure*" quality domain defined for article summaries (CSS) on a representative panel (n>=8) of conference highlights **(score x1)**: **/25**
- Apply scoring for the 2 items of the "*scientific and didactic value*" quality domain defined for article summaries (CSS) on a representative panel (n>=8) of conference highlights **(score x4): /40**

⇨ **Subscore scientific and didactic value (mean score from the individual abstracts analyzed): /65**

Total score COH (overall quality): /100

3.2.3. FAL quality score

No specific measurement scoring system has been developed for FALs which are licensed content that already went through a peer-review process and are selected among a defined set of journals in Neuroscience/Neurology. BHS quality review stakeholders will however address whether the following quality criteria are met based on the analysis of the FAL set delivered on a monthly basis:

- Currency of articles: Neurodiem publication date within one month from the original publication date;
- Balance between the 18 Neurodiem topics (sub-specialties/areas of interest).

3.2.4. IDS and Talks quality score

Neurodiem content scientific quality criteria and scoring system for original content, i.e. IDS and Talks are described below. 3 main quality domains "Credibility and relevance", "Scientific value and didactic dimension", "Reliability and format" have been defined. Each of them includes quality items which are rated on a 5-point scale according to achievement of the described criteria. Scores in the individual sections are summed to generate a sub-score related to each quality domain which is further weighted by a coefficient (indicated on the right upper hand side of the criteria grid). All sub-scores are summed to generate a total score of up to 100 corresponding to the overall quality of the IDS or Talks (scientific presentation or interview).
Table 3 provided in the appendix corresponds to the template of the scoring table used by reviewers when analyzing IDS and Talks scientific & medical quality.

Credibility and relevance **(score x3)**

Authors/speakers

Does the author/speaker meet quality standards in terms of academic seniority, reputation among peers in Neurology/Neuroscience, experience and recognized publications in the topic/field addressed in the IDS/Talks?

1- Strongly disagree
2- Disagree
3- Neutral
4- Agree
5- Strongly agree

Topic

1- Topic not related to the Neurology/Neuroscience field (off topic)
2- Topic in the neuroscience field, but not of specific interest for neurologists (poor contribution and impact in the field, or purely basic research issue with no interest to clinicians)
3- Topic being a relevant contribution in Neurology, but very far from any application in the clinical setting (e.g. advances in neurological disorders pathophysiologic mechanisms only)
4- Topic being a relevant and major contribution in the field, anticipated impact in the clinical practice as per author's conclusion
5- Topic being a relevant and major contribution in the field, with direct/immediate impact in the clinical practice as per author's conclusion

⇨ **Subscore credibility and relevance: /30**

Scientific and didactic value **(score x4)**

Structure

Does the structure of the article/presentation include the following sections over a logical development: i) introduction (background information, topic rationale and interest, perspective on the developed items); ii) key data (scientific evidence); iii) Scientific & medical novelties brought by the described findings and anticipated clinical impact; iv) Conclusion including summary of take-home messages?

1- Strongly disagree
2- Disagree
3- Neutral
4- Agree
5- Strongly agree

Accuracy and didactic dimension

Does the article/video provide sufficient background information and a relevant selection of scientific evidence and illustrations?

1- Strongly disagree
2- Disagree
3- Neutral
4- Agree
5- Strongly agree

Writing/speech quality

Does the article/presentation present the following items: clarity and coherence; ability to synthesize ideas and simplify concept; use of a neutral and factual tone; appropriate use of English/local language usage and grammar?

1- Strongly disagree
2- Disagree
3- Neutral
4- Agree
5- Strongly agree

⇨ **Subscore scientific and didactic value: /60**

Reliability (references) and format **(score x1)**

References

Does citations meet specifications in terms of: i) reliability (high-impact factor/recognized peer-reviewed journal articles or validated and up-to-date clinical guidelines; ii) number (IDS recommended range: (5-15 references))

1- Strongly disagree
2- Disagree
3- Neutral
4- Agree
5- Strongly agree

Format

- ***IDS***

Does IDS format respect the target length of 1000-1500 words?

1- Strongly disagree (<700 words or >3000 words)
2- Disagree (700-800 words or 2500-3000 words)
3- Neutral (800-900 words or 2000-2500 words)
4- Agree (900-1000 words or 1700-2000 words)
5- Strongly agree (1000-1700 words)

Or

- ***Scientific presentation***

Does the Scientific presentation respect the target duration of 5-10 min?

1- Strongly disagree (>16 min)
2- Disagree (14-16 min)
3- Neutral (12-14 min)
4- Agree (10-12 min)
5- Strongly agree (5-10 min)

Or

- ***Interview***

Does the interview respect the target duration of 5 min maximum?

1- Strongly disagree (>11 min)
2- Disagree (9-11 min)
3- Neutral (7-9 min)
4- Agree (5-7 min)
5- Strongly agree (3-5 min)

⇨ **Subscore reliability (references) and format: /10**

Total score expert points of view (IDS)/Talks (overall quality): /100

Appendix

Table 1: Template of scoring table supporting CSS scientific and medical quality assessment.

	**CSS –** *title – Link to Neurodiem webpage*		
**Criteria grid**	**Comments**	**Scale**	**Coefficient**
**Quality domain 1 - Reliability and relevance**		1-5	1
Journal			
Topic			
**Sub-score 1**		/10	**/10**
**Quality domain 2 - Structure**		1-5	2
Title and teaser text			
Take away			
Why this matters			
Study design			
Key results			
**Sub-score 2**		/25	**/50**
**Quality domain 3 - scientific and didactic value**		1-5	4
Accuracy			
Didactic dimension			
**Sub-score 3**		/10	**/40**
**TOTAL SCORE CSS – Overall quality**			**/100**

Table 2: Template of scoring table supporting COH scientific and medical quality assessment.
(A): Quality domain 1 - Congress selection relevance; (B): Quality domain 2- Congress coverage (topics); (C): Quality domain 3- Scientific and didactic value (individual abstracts)

A

	**COH –** *Name (acronym) of the congress +year – Link to congress homepage on Neurodiem*		
**Criteria grid**	**Comments**	**Scale**	**Coefficient**
**Congress selection relevance**		1-5	1
**Sub-score 1**		/5	**/5**

B

	**COH –** *Name (acronym) of the congress + year – Link to congress homepage on Neurodiem*		
**Criteria grid**	**Comments**	**Scale**	**Coefficient**
**Congress coverage**		1-5	2
Poster vs oral communications			
Scientific vs clinical-oriented topics			
“Hot topics”, “scientific/clinical highlights”, “late breaking news” sessions			
**Sub-score 2**		/15	**/30**

C

	**COH –** *Title of the abstract – Link to the Neurodiem webpage*		
**Criteria grid**	**Comments**	**Scale**	**Coefficient**
**Structure**		1-5	1
Title and teaser text			
Take away			
Why this matters			
Study design			
Key results			
**Score structure**		/25	**/25**
**Scientific and didactic value**		1-5	4
Accuracy			
Didactic dimension			
**Score accuracy + didactic dimension**		/10	**/40**
**Sub-score 3**			**/65**

⇨ **Total COH quality score (for one congress) up to a score of 100 = sub-score 1 (/5) + sub-score 2 (/30) + mean sub-score 3 (/65) for a representative panel of abstracts (n>=8) analyzed at individual level**

Table 3: Template of scoring table supporting IDS (A) and Talks (B) scientific and medical quality assessment.

A

	**IDS –** *Author and IDS title – Link to Neurodiem webpage*		
**Criteria grid**	**Comments**	**Scale**	**Coefficient**
**Quality domain 1 - Credibility and relevance**		1-5	3
Author			
Topic			
**Sub-score 1**		/10	**/30**
**Quality domain 2 - scientific and didactic value**		1-5	4
Structure			
Accuracy and didactic dimension			
Writing quality			
**Sub-score 2**		/15	**/60**
**Quality domain 3 - Reliability and format**		1-5	1
References			
Format (word numbers)			
**Sub-score 3**		/10	**/10**
**TOTAL SCORE IDS – Overall quality**			**/100**

B

	**Video –** *Speaker and video's title – Link to Neurodiem webpage*		
**Criteria grid**	**Comments**	**Scale**	**Coefficient**
**Quality domain 1 - Credibility and relevance**		1-5	3
Speaker			
Topic			
**Sub-score 1**		/10	**/30**
**Quality domain 2 - Scientific and didactic value**		1-5	4
Structure			
Accuracy and didactic dimension			
Speech quality			
**Sub-score 2**		/15	**/60**
**Quality domain 3 - Reliability and format**		1-5	1
References			
Format (video's duration)			
**Sub-score 3**		/10	**/10**
**TOTAL SCORE Video – Overall quality**			**/100**
